# DFT Simulations of the Vibrational Spectrum and Hydrogen Bonds of Ice XIV

**DOI:** 10.3390/molecules23071781

**Published:** 2018-07-19

**Authors:** Kai Zhang, Peng Zhang, Ze-Ren Wang, Xu-Liang Zhu, Ying-Bo Lu, Cheng-Bo Guan, Yanhui Li

**Affiliations:** School of Space Science and Physics, Shandong University, Weihai 264209, China; 17862711589@163.com (K.Z.); wangzeren96@163.com (Z.-R.W.); zhuxuliang@outlook.com (X.-L.Z.); lyb@sdu.edu.cn (Y.-B.L.); guancb@sdu.edu.cn (C.-B.G.); yhuili@sdu.edu.cn (Y.L.)

**Keywords:** ice XIV, vibrational spectrum, hydrogen bond, CASTEP, first-principles, DFT

## Abstract

It is always a difficult task to assign the peaks recorded from a vibrational spectrum. Herein, we explored a new pathway of density functional theory (DFT) simulation to present three kinds of spectra of ice XIV that can be referenced as inelastic neutron scattering (INS), infrared (IR), and Raman experimental spectrum. The INS spectrum is proportional to the phonon density of states (PDOS) while the photon scattering signals reflect the normal vibration frequencies near the Brillouin zone (BZ) center. Based on good agreements with the experimental data, we identified the relative frequency and made scientific assignments through normal vibration modes analysis. The two hydrogen bond (H-bond) peaks among the ice phases from INS were discussed and the dynamic process of the H-bond vibrations was found to be classified into two basic modes. We deduced that two H-bond modes are a general rule among the ice family and more studies are ongoing to investigate this subject.

## 1. Introduction

Water is not only an essential resource for the survival of all life, but also the most important part of living organisms. The solid state of water, ice, has been discovered in more than 19 different crystal phases and amorphous structures under certain pressure and temperature [1,2,3,4,5,6,7,8,9,10,11]. Usually, they are paired of hydrogen-ordered and hydrogen-disordered and can transform each other at a specific ambient condition. To facilitate the study of the normal vibration modes of ice, a hydrogen-ordered structure is beneficial to calculation and present the main physical features due to its simple primitive cell [12].

In 1998, Lobban et al. first reported the identification of a new ice phase named ice XII that crystallized at 260 K and 0.55 GPa [7]. In 2006, Salzmann et al. found that ice XII doped with HCl transformed into a new hydrogen-ordered phase upon cooling under pressure, which was called ice XIV with space group *P*2_1_2_1_2_1_ symmetry [8,13]. Since then, the Raman spectrum and inelastic neutron scattering of ice XIV/XII have been reported. Using the Raman spectrum, Salzmann et al. demonstrated the reversibility of the hydrogen order to disorder phase transition [14]. It also indicated that the new phase reported by Chou et al. [15] is in fact ice XII [16]. Yoshimura et al. measured in situ Raman spectrum of ice XII transformed from high density amorphous (HDA) to improve the understanding of the stability limits of ice XII [17]. Koza et al. studied the formation of ice XII under different conditions with a combined elastic and inelastic neutron scattering technique [18]. Later, Koza and coworkers presented the vibrational spectrum and identified the characteristic features compared with ice Ic [19].

Despite the many references presenting experimental vibrational spectra of ice XIV/XII, a theoretical method to assign the peaks was still lacking in reports until now. Herein, we calculated the most stable XIV structure using the first-principles density functional theory (DFT) to manifest the vibrational spectrum assisted by normal vibrational modes analysis.

## 2. Computational Methodology

Based on the DFT method of quantum mechanics, the CASTEP [20] code was used to perform geometric optimization and phonons calculation on the structure of ice XIV with the exchange-correlation (XC) function of generalized gradient approximation (GGA) RPBE [21]. It was reported by Brandenburg et al. that the geometric optimization of ice phases with PBE is better than that of RPBE [22], which was consistent with our test. However, the comparison between phonons calculation of PBE and experiment was very poor, far worse than the result of RPBE. Furthermore, the simulated vibration modes are constant regardless of the XC function. Thus, we preferred XC function of RPBE for this work.

The order of magnitude of energy and self-consistent field (SCF) tolerance were set as 1 × 10^−9^ for energy calculation. The energy cutoff was 750 eV, and the K-point grid was 2 × 2 × 3 in the reduced Brillouin zone (BZ). The norm-conserving pseudopotentials were used to calculate the PDOS and polarizability. The environmental pressure was set as 0.55 GPa. The phonons were calculated including LO-TO splitting. In addition, the option of Raman intensities was selected for the IR, Raman spectra calculation.

## 3. Results and Discussion

**Figure 1:**
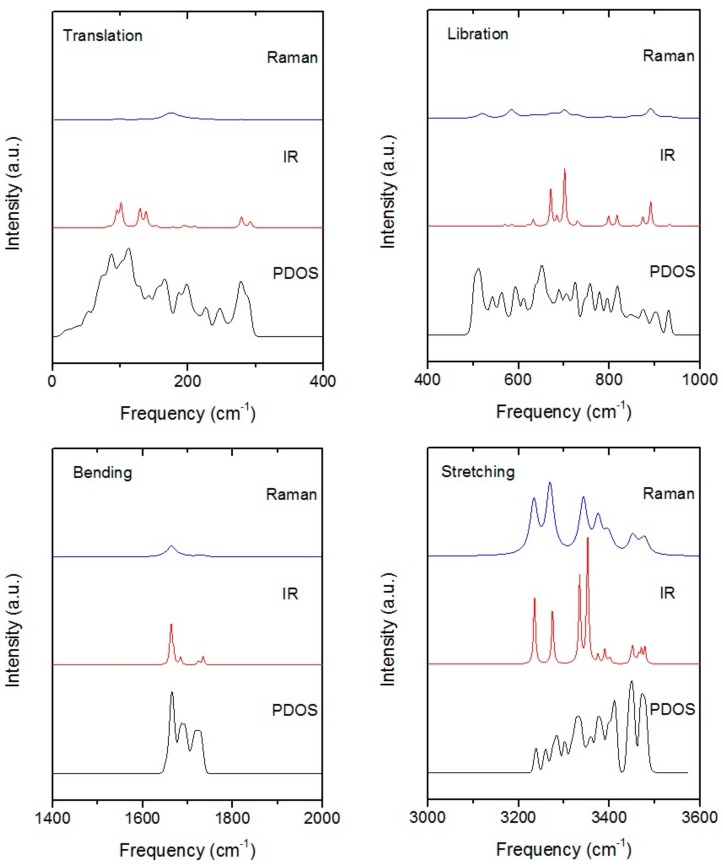
The simulated spectrum of ice XIV. The four images correspond to four vibration bands: translation, libration, bending, and stretching. Each image from top to bottom is Raman, IR, and the PDOS spectrum, respectively. Weak peaks were amplified reasonably.

The computational spectra of Raman, IR, and PDOS are ranked in Figure 1 by four separate bands. As the spectrum intensities of Raman and IR have quite a large scale, the relative proportions of two spectra in each band were adjusted for comparisons. The PDOS spectrum as well as normal vibration frequencies were calculated after precise geometry optimization. In theory, the spectrum by inelastic neutron scattering (INS), is proportional to PDOS, whilst the photon scattering can only collect signals near the BZ center. Table 1 presents the comparisons of the PDOS against neutron scattering, and the normal vibration frequencies against Raman scattering. Since ice XIV is a hydrogen-ordered phase of ice XII, the spectrum of XIV showed more pronounced features than XII. Both of the Raman spectra of ice XIV and XII could then be used for peaks assignment. There are 12 molecules in a primitive cell of ice XIV. Thus, the number of optic normal modes was 12 × 3 × 3 − 3 = 105. Each band of the spectrum is discussed in detail as follows.

**Figure 2:**
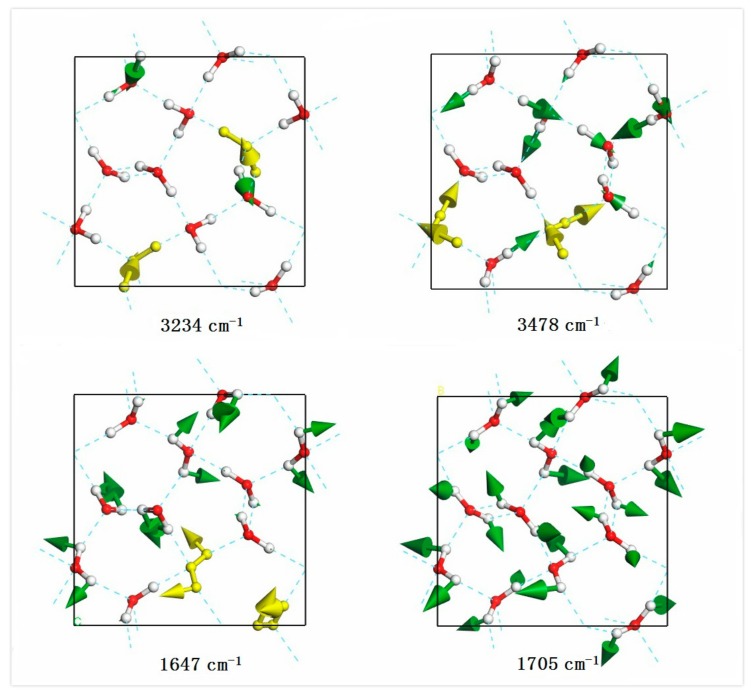
A top view of four normal vibration modes in the stretching band (3234 cm^−1^, 3478 cm^−1^) and bending band (1647 cm^−1^, 1705 cm^−1^), respectively. Typical vibrations are represented in gold. The green arrows represent the vibration direction in sizes proportional to the vibration amplitude.

The stretching band is the region of O–H covalent bond stretching. There are 24 normal vibration modes and the frequencies of mode range from 3234 cm^−1^ to 3478 cm^−1^. Each molecule has two kinds of vibration: symmetric stretching and asymmetric stretching. As shown in Figure 2, there are two molecular vibration images corresponding to the minimum and maximum vibration frequencies, respectively. This is a top view and the green arrows represent the vibration direction in sizes proportional to the vibration amplitude. For the minimum mode at 3234 cm^−1^, four molecules presented symmetric stretching vibration; two examples are illustrated in gold. It has been found for ice XV that some modes include the isolated vibration of only one O–H bond while the other one does not vibrate [23]. This was a similar case in ice XIV such as the mode at 3234 cm^−1^ illustrated in Figure 1. We attributed this exotic phenomenon to geometry deformation of the local tetrahedron structure under pressure. Almost all molecules showed asymmetric stretching at 3478 cm^−1^, which was consistent with the literature where energy increases from symmetric stretching to asymmetric stretching. To maintain a static center of mass, the vibrations of the molecule are correlated in a primitive cell. An obvious phenomenon was that the Raman and IR spectrum were more sensitive to symmetric stretching. From the spectrum of Figure 1, one can see several distinct peaks in the symmetric stretching region due to the restrictions of selection rules.

Salzmann et al. recorded the Raman spectrum of recovered hydrogen-ordered ice XIV and identified the vibration modes by the deuterated method [14]. By comparison, they detected six Raman peaks at 3214 cm^−1^, 3317 cm^−1^, 3326 cm^−1^, 3346 cm^−1^, 3368 cm^−1^, and ~3407 cm^−1^, which had good agreement with this study at 3234 cm^−1^, 3269 cm^−1^, 3343 cm^−1^, 3375 cm^−1^, 3395 cm^−1^, and 3451 cm^−1^ (Table 1). They reported that the peak at 2480 cm^−1^ of D_2_O was not observed at 2480 × 1.35 = 3348 cm^−1^ in H_2_O of ice XIV. According to Figure 1, we found that the peak should be at 3377 cm^−1^ (normal mode). Salzmann et al. also performed a Raman spectrum of ice XII [16], where peaks at 3209 cm^−1^, 3310 cm^−1^, 3340 cm^−1^, and 3415 cm^−1^ were consistent with normal mode at 3234 cm^−1^, 3269 cm^−1^, 3375 cm^−1^, 3451 cm^−1^, respectively.

In the intramolecular bending vibration band, there were 12 normal vibration modes ranging from 1647 cm^−1^ to 1735 cm^−1^. Considering a collective vibration, all the bending modes can be classified as in-phase and out-of-phase vibrations. Figure 2 shows two bending vibration images at frequencies of 1647 cm^−1^ and 1705 cm^−1^. The molecules in gold at 1647 cm^−1^ showed a pair of out-of-phase modes. According to our previous work, we found that the energy increasing trend was from in-phase bending to out-of-phase bending. However, for the case of ice XIV, the lowest mode at 1647 cm^−1^ showed four pairs of out-of-phase vibrations. The vibration mode at 1705 cm^−1^ showed the in-phase vibrating of all molecules. Although the simulated Raman and IR spectrum both contained a peak at 1663 cm^−1^, the intensity was too weak to be detected in IR.

**Figure 3:**
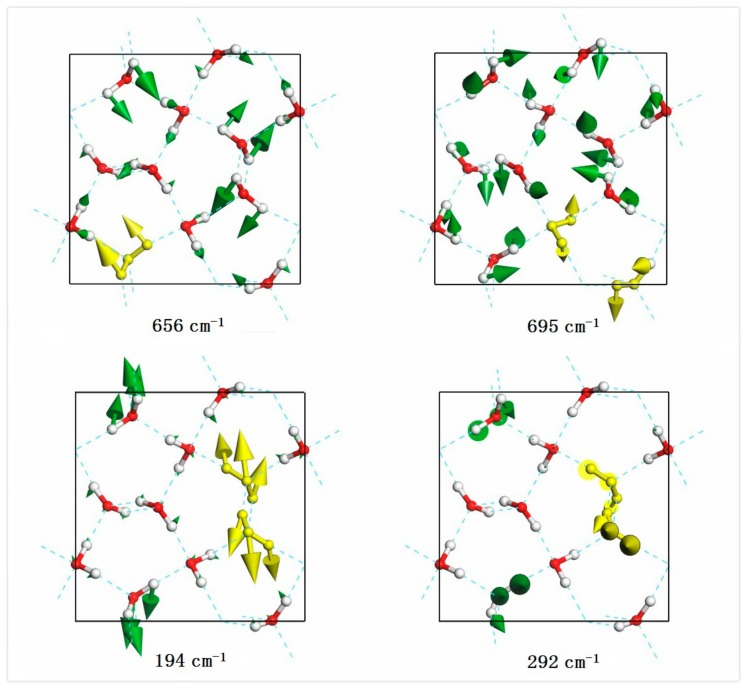
A top view of the four normal vibration modes in the libration band (656 cm^−1^, 695 cm^−1^) and translation band (194 cm^−1^, 291 cm^−1^), respectively. For the dynamic process of weak and strong H-bond, please see the Supplementary Videos S1 and S2 (194.mp4 and 292.mp4).

As for the intermolecular librational band, there were 36 normal modes and the frequencies ranged from 502 cm^−1^ to 933 cm^−1^. In this region, all the molecular vibration modes could be classified into three types: rocking, twisting, and wagging. A rocking mode is oscillating in plane while a wagging is oscillating perpendicular to the molecular plane. A twisting mode is a rotation around the bisector of angle. For the mode at 656 cm^−1^ in Figure 3, the highlighted molecule showed wagging vibration while the 695 cm^−1^ mode presented combinations of rocking and twisting. Interestingly, we found that all molecules were rocking at 523 cm^−1^ and all molecules were wagging at 933 cm^−1^, which meant that the energy of rocking was lower than wagging. From the simulated Raman spectrum, we could only see four weak peaks and the IR spectrum had three sharp peaks at 671 cm^−1^, 701 cm^−1^, and 890 cm^−1^, which were combinations of the three vibration types.

Compared with the experimental data, six vibration peaks (462 cm^−1^, 488 cm^−1^, 527 cm^−1^, 562 cm^−1^, 790 cm^−1^, and 867 cm^−1^) from Raman scattering [14] were in agreement with normal mode calculations (518 cm^−1^, 524 cm^−1^, 583 cm^−1^, 585 cm^−1^, 798 cm^−1^, and 891 cm^−1^). However, a peak recorded at 413 cm^−1^ could not be assigned from the simulations. We suspected that this was a false peak according to the DFT simulations. The peak at 490 cm^−1^ (Reference [15])/470 cm^−1^ (Reference [16]) should be the frequency at 518 cm^−1^ (normal mode in Table 1). As for the INS spectrum, we could roughly match the peaks of ~464 cm^−1^, ~560 cm^−1^, ~632 cm^−1^, ~760 cm^−1^, ~904 cm^−1^ (Reference [18]) to 504 cm^−1^, 563 cm^−1^, 639 cm^−1^, 757 cm^−1^, and 903 cm^−1^ (PDOS).

In the intermolecular translational band, there were 33 normal vibration modes ranging from 62 cm^−1^ to 292 cm^−1^. For one molecule, there existed three vibration modes: stretching, wagging, and rocking. Note that, rocking in the translational band is a monolithic movement while rocking in the librational band is only hydrogen vibration within one molecule. To date, the physical mechanism of the H-bond interaction is still in question [23,24,25,26]. Li et al. proposed a two-strength model based on electrostatic interactions to explain the two main peaks from the INS spectrum [27,28]. The model has not been widely accepted so is still a controversial topic [29,30,31,32]. He et al. disagreed with this model and asserted that these two peaks originated from the different directionalities of motion manifested in the distinct polarizations of the absorbed photons [32]. In our previous study, we found that the two H-bond peaks in the hydrogen-ordered ice Ic originated from two different H-bond vibration modes. There are four H-bonds linked with one molecule to form a tetrahedral structure in ice crystals. For ice Ic, there are two kinds of vibration modes for one molecule: two H-bonds vibrating mode and four H-bonds vibrating mode with a strength ratio of $\sqrt{2}$ [33]. These two types of modes were also observed in the ice XIV crystal lattice. In the translational band of Figure 1, the peak of maximum strength observed at 288 cm^−1^ was very distinct. Phonons at above ~200 cm^−1^ were the main four H-bond vibrations analyzed by normal vibration modes. The example of the strong mode at 292 cm^−1^ in Figure 3 shows that molecules were stretched along its angle bisector. This is a typical four H-bonds vibration mode where four linked H-bond are oscillating together. The two H-bonds mode means that one molecule vibrates towards two connected neighbors where there are two effective H-bonds oscillating for this mode. Subject to the local tetrahedral structure, the collective vibrations are combinations of wagging and rocking as illustrated in Figure 3 of the weak mode at 194 cm^−1^. To show the dynamic process of these two modes, please see the videos from the Supplementary Files. For ice XIV, the local structure could not maintain a regular tetrahedron due to lattice deformation under pressure. Thus, deviation strength ratios from $\sqrt{2}$ were reasonable. From Figure 1, it can be seen that there were two main peaks at 166 cm^−1^ and 199 cm^−1^. Phonons around this region were mainly two H-bond modes. Other vibration modes were associated with cluster vibration or skeleton deformation below 166 cm^−1^. These optic phonons were merged with acoustic phonons.

In the simulated Raman spectrum of Figure 1, there was a small peak at 176 cm^−1^, which should correspond to experimental observations at 192/195/214 cm^−1^ [14,15,16]. The red-shift was mainly due to the RPBE function underestimating for the H-bond [13]. As for the INS spectrum, Koza et al. mentioned several peaks in the far-IR region such as 7 meV, 10 meV, 12 meV, and 24 meV (56 cm^−1^, 80 cm^−1^, 96 cm^−1^, and 192 cm^−1^) [18] and could be matched with 53 cm^−1^, 75 cm^−1^, 88 cm^−1^, and 188 cm^−1^ (PDOS in Table 1). Two distinct peaks at 26 meV and 36 meV (208 cm^−1^ and 288 cm^−1^) [19] were in agreement with 204 cm^−1^ and 288 cm^−1^ (PDOS).

## 4. Conclusions

In summary, using the first-principles DFT method, we presented the Raman scattering, IR absorption, and the INS spectrum (PDOS) of ice XIV, theoretically. The PDOS spectrum was used to assign the characteristic peaks in the INS spectrum as the INS collects signals throughout the whole BZ without selection so that the simulated PDOS spectrum is proportional to the INS. The 105 optic normal vibration modes in the BZ center could be compared with the Raman and IR spectra. Under this condition, the peaks recorded from photon scattering could be assigned according to normal vibration mode individually.

The most valuable result was the identification of two types of H-bond vibration modes. The thermodynamic nature of the H-bond is a fascinating, but still ambiguous topic. Subject to molecular conformation, there are two types of O–H stretching modes, i.e., symmetry and asymmetry stretching and one bending mode in the intramolecular vibrational region. In the librational band, the vibration mode for a molecule is one of rocking, wagging, and twisting. As for the translational band, it is hard to analyze the H-bond vibration due to the complexity of lattice collective motion. Inspired by previous study on ice Ic, we found that there were also two basic H-bond vibration modes for one molecule in ice XIV. Deviations mainly resulted from structural deformation of the local regular tetrahedron under pressure. Considering the local structure symmetry, we deduced that two H-bond modes were a general rule among the ice family and more studies are ongoing to investigate this subject.

## Figures and Tables

**Table 1:** Comparison of calculated results with Raman and INS data. The main peaks of PDOS in the first column are compared against INS spectrum. The frequencies of 105 normal vibration modes with Raman intensities are compared against the experimental Raman peaks.

PDOS	Neutr. Scattering (References [18]/[19])	Normal Modes	Raman Intensity	Raman Scattering (References [14]/[15]/[16])
53	56/—	62	0.01	
75	80/—	82	0.00	
		83	0.03	
		84	0.11	
88	96/—	90	0.00	
		92	0.00	
		94	0.17	
		95	0.06	
104		100	0.08	
		101	0.10	
		102	0.01	
113		104	0.03	
128		127	0.00	
		128	0.04	
		129	0.00	
142		138	0.18	
		153	0.01	
159	160/—	154	0.02	
166		162	0.00	
		169	2.21	
		176	1.70	
		178	2.42	214/192/195
188	192/—	192	0.93	
		193	0.11	
		194	0.01	
199	—/~208	199	0.16	
		209	0.07	
226		211	0.67	241/—/—
248		236	0.14	
		277	0.17	314/—/—
279		279	0.16	
288	—/~288	289	0.00	
		292	0.08	
504	~464/—	502	0.39	
512		510	0.06	
		518	3.88	462/490/470
		523	0.45	
		524	1.23	488/—/—
543		545	0.01	
563	~560/—	570	0.25	
		583	4.54	527/—/—
		585	4.34	562/—/553
593		591	1.13	
612		613	0.55	
		620	0.01	
		625	1.51	
		631	0.20	
		632	0.61	
639	~632/—	642	0.09	
		643	1.56	
652		657	1.41	
		671	3.72	
688		684	1.34	
		695	0.05	
		701	6.14	
705		702	2.20	
725		724	1.10	
		729	1.14	
748		733	1.30	
757	~760/—	751	0.03	
777		790	0.42	
796		798	1.50	790/—/—
817		817	0.49	
846		845	0.43	
		853	2.00	
876		874	0.51	
903	~904/—	891	10.72	867/—/—
930		932	0.00	
		933	1.50	
		1647	1.24	
		1661	4.30	
1665		1663	5.87	
		1668	1.15	
		1669	1.42	
		1671	0.04	
1687		1684	1.46	
1693		1705	0.32	
1720		1722	0.01	
1727		1725	1.05	
		1734	0.10	
		1735	0.80	
		3234	1519.40	3214/3215/3209
3238		3235	15.13	
3260		3269	1975.96	3317/—/3310
3283		3275	22.23	
3302		3322	1.01	
3333		3334	72.87	
		3343	1510.66	3326/—/—
		3351	0.17	
3358		3352	21.79	
		3375	931.33	3346/—/3340
3376		3377	37.50	~3348/—/—
		3390	26.01	
		3395	343.97	3368/—/—
3400		3398	41.58	
		3402	170.77	
3411		3411	31.95	
		3447	0.61	
3449		3450	77.66	
		3451	434.71	~3407/3410/3415
		3458	12.08	
		3465	38.95	
		3467	17.76	
3473		3470	104.38	
		3478	396.50	

## Supplementary Materials

The following are available online, Video S1: Dynamic process of H-bond vibration at 292 cm^−1^. Video S2: Dynamic process of H-bond vibration at 194 cm^−1^.
